# Foraging Behavior of the Dead Leaf Butterfly, *Kallima inachus*

**DOI:** 10.1673/031.013.5801

**Published:** 2013-06-21

**Authors:** Yuchong Tang, Chengli Zhou, Xiaoming Chen, Hua Zheng

**Affiliations:** 1 Key Laboratory of Cultivation and Utilization of Resource Insects of State Forestry Administration; Research Institute of Resource Insects, Chinese Academy of Forestry, Kunming, Yunnan, China

**Keywords:** behavioral tests, color selection, EAG responses, fruit feeding, foraging adults, volatile compounds

## Abstract

The behavioral responses of foraging adults of *Kallima inachus* (Boisduval) (Lepidoptera: Nymphalidae) to four colors and to six different fermented fruit juices were observed in order to determine the cues used by foraging adults. According to the results, adults did not show a behavioral response to red, yellow, purple, or white artificial flowers without food odors, but flowers with the fermented pear juice strongly attracted them, and they showed a behavioral response to fermented juices of the six fruits (pear, apple, banana, watermelon, orange, and persimmon) with no statistically significant preference. The fruit volatiles were collected using dynamic headspace adsorption, and the volatile components were analyzed by auto thermal-desorption gas chromatography-mass spectrometry to assess which volatiles existed in the fruits. Only alcohols, esters, and ketones were common in the volatiles of all six fermenting fruits. The five volatile components found in the six fruits, as well as two others found to be in other fermented foods by previous studies, were selected to test the behavioral and electroantennogram (EAG) responses of naive adults to estimate behavioral preference and antennal perception. In field behavioral tests, alcohols were the most attractive, followed by esters, while α-pinene, butanone, and acetic acid were much less attractive. Relative to other volatile combinations and ethanol alone, the mixture of ethyl acetate and ethanol attracted the most feeding adults. The number of adults attracted was significantly positively correlated with the concentration of both ethanol and ethyl acetate. The EAG responses of naive adults showed that the EAG responses to 3-methyl-1-butanol, isoamyl acetate, ethyl acetate, α-pinene, butanone, and acetic acid were all higher than those to ethanol (100%) at doses of either 5 µl/mL or 50 µl/mL. Sexual differences only existed in 3-methyl-1-butanol and acetic acid at particular concentrations. Sexual differences in response to chemical mixtures were not significant at 50 µl/mL. In addition, the EAG responses in the within-sex trials were not correlated to the dosage (0.01, 0.1, 1, 5, 10, and µl/mL) of either ethanol or ethyl acetate. The results showed that olfactory cues played a crucial role in the foraging of adult *K. inachus*, and that foraging adults can use a variety of chemical signals derived from food; however, the feeding preference to volatiles was not necessary correlated with the EAG responses.

## Introduction

To maintain daily life activities and reproduction, adult insects must look for and find suitable nutrients to supplement their diets. Insects are known to make use of a variety of sensory modalities in foraging, and the integration of visual, olfactory, and gustatory cues are usually involved in their orientation to and finding of food sources (Barth 1991).

Depending on the food type, adult butterflies can be classified into three guilds: nectar-feeding, fruit-feeding, and a combination of both (Gilbert 1972; DeVries 1988; DeVries et al. 1997; DeVries and Walla 2001; Molleman et al. 2005; Ômura and Honda 2009). Most butterfly adults are found in temperate regions and feed on nectar, while fruit-feeding butterflies are found mainly in tropical and subtropical regions and feed on rotting fruits, exuded tree sap, mud, carrion, and dung (Boggs and Jackson 1991; Ômura et al. 2000; DeVries and Walla 2001; Krenn 2008).

The scientific literature clearly demonstrates that the flower-visiting behavior of adult butterflies is affected by the color and odor of flowers, which provide specific information for adults, and that colors are extremely important in flower recognition and preference in some species (Use and Vaidya 1956; Weiss 1997; Andersson and Dobson 2003; Borges et al. 2003). Flower scent is an olfactory stimulus that influences butterfly visitation (Andersson 2006; Ômura 2006). For example, 2-phenylethanol and phenylacetaldehyde stimulate the foraging behavior of *Pieris rapae* (Honda et al. 1998; ômura et al. 1999a,b; ômura and Honda 2005).

In fruit-feeding butterflies, most studies have focused on the effects of adult diet quality on longevity, reproductive output, and egg-hatching success (Braby and Jones 1995; Bauerfeind and Fischer 2005; Bauerfeind et al. 2007; Geister et al. 2008; Molleman et al. 2008). However, a few studies have investigated the foraging mechanism of saprophagous butterflies, including the behavioral responses of *Kaniska canace* and *Vanessa indica* to the exuded tree sap of *Quercus* spp. (Fagaceae) (ômura et al. 2000). Ethanol and acetic acid, together with sugars at low concentrations, synergistically stimulated butterfly feeding behavior and enhanced probing responses in *K. canace* and *V. indica* (ômura and Honda 2003). ômura and Honda (2009) studied the behavioral and electroantennographic responses of adult butterflies of *K. canace, V. indica,* and *Nymphalis xanthomelas* to stimuli. Moreover, Dierks and Fischer (2008) considered that, in the tropical butterfly *Bicyclus anynana*, only sugars and alcohols acted as feeding stimuli, while alcohols functioned primarily as long-range signals, guiding the butterflies to food sources. Compared with flowers, decayed food provides severely limited visual cues to attract fruit-feeding insects, thus odor seems to serve as the pivotal cue in their foraging (Ômura et al. 2000).

*Kallima inachus* (Boisduval) (Lepidoptera: Nymphalidae) is a well-known mimetic insect, is distributed in tropical and subtropical regions of eastern and southern Asia, and mainly lives in broad-leaved forests at altitudes of 500–1200 m.a.s.l. In natural conditions, adults take exuded tree sap, decayed fruits, and dung as supplementary nutrients, but are also reported to occasionally visit flowers (Igarashi and Fukuda 2000). In many areas, wild populations have sharply declined with increased habitat destruction and fragmentation. Understanding the adult feeding habits and food information mechanism will help in developing habitat conservation measures for *K. inachus*. To date, the effects of olfactory stimuli of food on adult butterflies have been studied with indoor proboscis extension reflex experiments, which evaluate responses to fixed odor sources in defined locations over very small distances, and the results may not fully reflect the actual responses of adults in wild habitats. In addition, how the researchers select trapping baits and the methods used for trapping may introduce bias in such investigations (Hughes et al. 1998). In this study, we observed the behavioral responses of foraging *K. inachus* adults to different colors of artificial flowers and to different fermented fruits in a large-scale net house. We collected fruit volatiles using dynamic headspace adsorption and analyzed them with auto thermal-desorption gas chromatography/mass spectrometry. Finally, combining these results, we tested butterfly feeding responses and electroantennogram (EAG) responses to volatile fractions to investigate (1) whether adult feeding was influenced by food color, (2) which kinds of volatiles that existed in the fermented fruits we tested could be used by the foragingadults, and (3) which food chemical signals could be perceived and preferred by the foraging adults.

## Materials and Methods

### Experimental arena

Experiments were performed at the Experimental Station of the Research Institute of Resource Insects of the Chinese Academy of Forestry in Yuanjiang, Yunnan (400 m.a.s.l., 101.59°–101.00° E, 23.35°–23.36°N). A net house (12 m long, 8 m wide, and 5 m tall) with evenly scattered sunlight was used as the experimental chamber.

### Study animals

Four-day-old naive adults of *K. inachus* were obtained from artificial culture. Larvae of *K. inachus* were reared on fresh leaves of *Strobilanthes cusia* (Acanthaceae) at 25°C and with a photoperiod of 14:10 L:D. Adults were supplied with purified water and were not allowed to contact fruit or sap before tests.

### Feeding responses to different colors of artificial flowers

The local common nectar flowers are *Asclepias curassavica* (Asclepiadaceae), *Lantana camara* (Verbenaceae), and *Parsonsia laevigata* (Apocynaceae). Their corolla colors are red-yellow, yellow-deep magenta, and white, respectively. So, red, yellow, purple, and white were selected as the colors to be tested. All the artificial flowers were made of cotton. The four colored artificial flowers with the same shape (D = 4.64 ± 0.08 cm) were used to assay butterfly response to flower color. The same colored flowers were put one by one in the same transparent glass disk, which was 1.5 cm in height and 22.5 cm in diameter. The disks with flowers were placed 80 cm apart in a row and 1.3 m above the floor in the center of the net house. The evening before the bioassay, about 200 adult butterflies (sex ratio not controlled) were released into the net house and allowed free-flight to acclimatize to the experimental arena. The number of feedings during two 2-hr periods (10:00–12:00 and 15:00–17:00) was recorded for each model flower bunch. During the bioassay, the positions of the different disks of colored flowers were rotated in a clockwise manner every hour. A total of four persons were standing 30 cm away from the samples to record frequency of feedings on the colored, cotton flowers. The observations were repeated as described on the second day. The data were analyzed with a chi-square test.

When an adult *K. inachus* landed nearby the food, and then reached to the food and uncoiled the proboscis to probe and feed, it was recorded as a visit.

### Feeding responses to different fermented fruit juices

The effects of odor on the feeding behavior of *K. inachus* were examined using fermented fruit juices. Commercially purchased fruits (pear, apple, banana, watermelon, orange, and persimmon) were sealed in plastic bags respectively and fermented outdoors at natural temperatures (25–32° C, 37.3–86.9% relative humidity) for four days prior to the experiments. After fermentation, we cut about 300 g of each kind of rotting fruit into small pieces, squeezed juice from each fruit, diluted the juice with 200 mL deionized water, and stored the resulting liquid at -10° C until use. Each model consisted of a glass disk (D = 22.5 cm) covered with red cloth in order to minimize differences in fruit juice color. Covered disks were placed in a row 1.3 m above the floor and 60 cm apart in the center of the net house. Ten percent ethanol dissolved in deionized water was used as a control, and deionized water was used as a blank control. The evening before the bioassay, about 250 adults (sex ratio not controlled) were released into the net house and allowed free-flight to acclimatize to the experimental arena. The number of feedings during two 2-hr periods (10:00–12:00 and 15:00–17:00) was recorded for each model. During the bioassay, the positions of the different juice disks were changed every half hour. The observations were repeated on a second day. The data were analyzed with a chi-square test.

### Feeding responses to different colored flowers with fermented pear juice

The cotton flowers were arranged the same as in the test of feeding responses to different colors of artificial flowers. Then, we sprayed fermented pear juice evenly on the petals. The juice that couldn't be absorbed by the cotton petals flowed into the disk. The evening before the bioassay, about 200 adult butterflies (sex ratio not controlled) were released into the net house and allowed free-flight to acclimatize them to the experimental arena. The number of feedings during two 2-hr periods (10:00–12:00 and 15:00–17:00) was recorded for each model. During the bioassay, the positions of the different juice disks were changed in a clockwise direction every hour, and the flowers and juice were renewed at the same times. The observations were repeated on a second day. The data were analyzed with a chi-square test.

### Collection and chemical analyses of volatile compounds from six different fermented fruits

The dynamic headspace adsorption method was used to collect volatile compounds from six different rotting fruits. The fruits were enclosed in polyacetate bags (406 mm × 444 mm, large size, Reynolds, http://www.reynoldskitchens.com/), and volatiles were drawn from the enclosure with a battery operated membrane pump for 40 min. Volatiles were trapped on cartridges containing the adsorbent Tenax-GR. Before use, the cartridges were cleaned with 2 mL diethyl ether, dried with nitrogen gas, then wrapped in aluminium foil and stored in polyacetate bags. The chemical compositions of fruit volatiles were examined by auto thermal-desorption gas chromatography/mass spectrometry using a Perkin Elmer Clarus 600 GC, Clarus 600T MS instrument, and a TurboMatrix 650 ATD as the thermal desorption unit. The carrier gas velocity was 2.0 mL/min with a two stage desorption. The Tenax GR tubes were heated at 260° C for 10 min, while the desorbed compounds were simultaneously collected into a cooling trap at -30° C. The cold trap was heated at a rate of 40° C/sec to a final temperature of 300° C for 5 min for the transfer of analytes to the gas chromatography column. The purge time was 1 min. The desorb flow rate was 2,520 mL/min. The flow rates of the inlet and outlet splits were 1,520 mL/min and 20 mL/min, respectively. Gas chromatography analyses were carried out with a gas Chromatograph on an Elite-5 MS capillary column (30 m × 0.32 mm, 0.25 µm, Agilent, http://agilent.com). The heating program was as follows: hold at 40° C for 2 min, increase by 6° C/min to 130° C, isothermal for 5 min, increase by 15° C/min to 280° C, then isothermal for 5 min. For mass spectrometry, the ion source temperature was 220° C, the interface temperature was 250° C, each scan lasted 0.2 sec with a recovery time of 0.1 sec, and the scan range (m/z) was 29–600. Identifications of compounds were made by comparing the obtained mass spectra and the total ion currency profile with those in a computer library NIST and the software TurboMass. In addition, a combination of classical gas chromatographic retention time data and chemical analysis experience were analyzed. The relative content of each compound was determined by using the peak area normalization method.

### Test compounds

Based on the results of auto thermal-desorption gas chromatography/mass spectrometry, we used seven pure chemical compounds in behavioral and EAG response tests. All seven chemicals were commercially purchased and were more than 98% pure. These compounds were 3-methyl-1-butanol, ethyl acetate, isoamyl acetate, α-pinene, the 2-pentanone homologue butanone, ethanol, and acetic acid. The first five compounds were present in at least three of the six kinds of fermented fruits; ethanol and acetic acid are the major volatiles of fermentation, but were not found in our fermented fruits.

### Behavioral responses to different volatiles

In our long-term field observation, we found *K. inachus* habitually basks on light colors. In order to avoid color interference, white disks (D = 20 cm) were covered with deep-purple cloth, spaced above the floor in the net house, and filled with 25 mL of a solution, which was dissolved in deionized water as described below. Deionized water was used as a control. Three feeding experiments were conducted:

1. In the center of the net house, a 6 m × 6 m square was delimited, and the four corners of the square and the midpoint of the four sides were chosen as the placement points of the treatments. The feeding performances of adult butterflies were characterized by assaying seven single compounds at concentrations of 5% v/v.
2. In the center of the net house, a 6 m × 3 m rectangle was delimited, and the four corners of the rectangle and the midpoint of the long sides were chosen as the placement points. The synergistic effects of different compound mixtures were evaluated. Based on the results of the single-compound assays, we mixed ethanol, α-pinene, and ethyl acetate at equal volumes in the following combinations: ethanol + ethyl acetate + α-pinene, ethanol + ethyl acetate, ethanol + α-pinene, ethyl acetate + αpinene, and ethanol. Each mixture had a total concentration of 5%.
3. In the center of the net house, a 6 m × 5 m rectangle was delimited, and the four corners of the rectangle and the one-third point of the four sides were chosen as the placement points. To identify the most effective doses of ethanol and ethyl acetate, a concentration series (0.001, 0.01, 0.1, 1, or 10%) of each compound was bioassayed; 0.5% ethanol was also used.

Before each of the three bioassays, about 100 adults (sex ratio not controlled) were released into the net house and allowed free-flight to acclimatize them to the experimental arena. The number of feedings during two 2-hr periods (09:30–11:30 and 14:00–16:00) was recorded for the treatment and control models. Every model was duplicated. During each bioassay, the positions of the models were rotated clockwise every 30 min, 40 min, or 20 min, respectively, and solutions were renewed to account for evaporation. Tests were replicated for two days. Significant differences between treatments and controls were determined using analysis of variance, followed by the LSD test.

### Electroantennogram recording

EAG bioassays were performed using the procedures described by Fadamiro et al. (2010). Antennal sensitivity to fruit volatiles or their homologues and to ethanol and acetic acid were measured with electroantennographs (Instrument: PRG-2, Syntech, http://www.syntech.nl/). Antennal preparations were made by first cutting the basal and distal ends of the antennae with a scalpel and mounting the antennae vertically on electrodes with conductive adhesive. The ether was taken as solvent to prepare the sample. A 100 µl aliquot of each solution (see below) was applied to a piece of filter paper (5 × 40 mm). After evaporating the solvent at room temperature, the filter paper was placed in a sample cartridge (6 mm ID × 50 mm). An odor puff was mixed with a humidified airstream blowing continuously over the antennae through 6-mm-ID glass tubing, which ended 1 cm from the antennal preparations. At least 30 sec were allowed between successive stimulations for antennal recovery; each stimulus lasted about 0.1 sec. Each antenna was tested five times, and each antenna originated from a different butterfly. When successive samples involved the same compound(s) at different concentrations, the test order was from lowest concentration to highest. Odorant stimuli were presented in a random order. Six males and six females were employed for the EAG recording for each compound or mixture, and the average response was calculated.

Four EAG experiments were performed:

1. The EAG responses to the solutions at doses of 5 µl/mL were tested. A series of odorant compounds were applied to butterfly antennal preparations in the following order: ether control, standard stimulus (see below), individual samples (3-methyl-1-butanol, butanone, αpinene, acetic acid, ethyl acetate, isoamyl acetate) in a random order, standard stimulus, and ether control. Each sample was tested five times in each antenna.
2. The EAG responses to the solutions at doses of 50 µl/mL were tested, with solutions and procedures as described above.
3. The EAG responses to mixtures at the dose of 50 µl/mL were tested. Mixtures mixed at equal volume were as described for the behavioral feeding experiments.
4. To identify the EAG responses to different doses of ethanol and ethyl acetate, ethanol was tested at six doses (0.01, 0.1, 1, 5, 10, or 100 µl/mL) and ethyl acetate was tested at five doses (0.01, 0.1, 1, 10, or 100 µl/mL).

**Figure 1.**
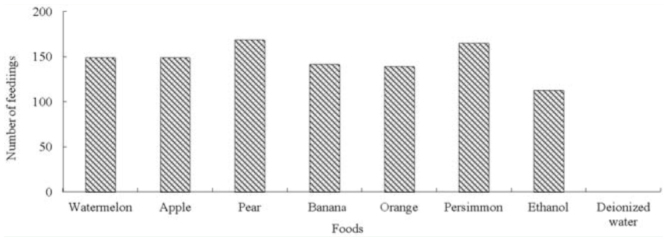
Frequency of feeding of *Kallima inachus* at different fermented fruit juices. High quality figures are available online.

**Figure 2.**
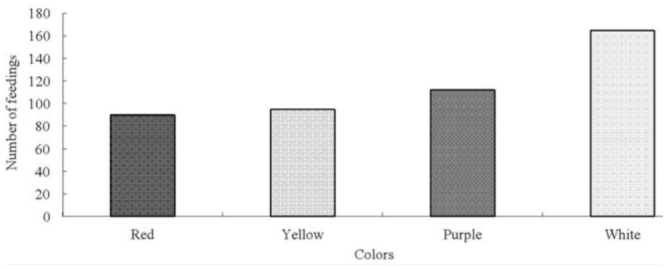
Frequency of feeding of *Kallima inachus* at different colored flowers supplemented with fermented pear juice. High quality figures are available online.

Ethanol at 5 µl/mL was used as the standard stimulus in EAG experiments 1 and 4, and 50 µl/mL was used in experiments 2 and 3. The responses were averaged across individual trials and expressed as percentages of the response relative to the standard. Data were normally distributed; sexual differences in EAG intensities were analyzed with a *t*-test. The intensity of EAG responses to different compounds within males and within females were analyzed using analysis of variance, followed by the LSD test.

## Results

### Responses to different colors of artificial flowers

*K. inachus* showed no attempted feeding or orientation responses to the four different single colors, suggesting that foraging adults were not innately sensitive to these colors, and that color is not a forging signal for *K. inachus*.

### Responses to different fermented fruit juices

*K. inachus* showed no visits to deionized water. The different fermented fruit juices were not significantly differently attractive to *K inachus* (chi-square test: χ^2^ = 4.895, *p* > 0.05), but the differences between the juices and ethanol were significant (χ^2^ = 14.053, *p* < 0.05). *K. inachus* showed the strongest response to fermented pear juice, accounting for 16.47% of feedings, while ethanol had the weakest effect, attracting only 11.01% of feedings (Figure 1).

### Responses to different colored flowers with fermented pear juice

When artificial flowers were supplemented hourly with fermented pear juice, the number of *K. inachus* feeding at all four flower colors increased dramatically, and color preference for white was significant (χ^2^ = 30.589, *p* < 0.01). Adults showed the strongest attraction to white, with 35.71% feeding at that color, and weaker attractions to red, yellow, and purple (19.48%, 20.56%, and 24.24% feeding, respectively). There were no differences in feeding among red, yellow, and purple flowers (χ^2^ = 2.687, *p* > 0.05) (Figure 2).

### Collection and chemical analyses of volatile compounds from six different fermented fruits

All six fruits contained alcohols, esters, and ketones, among their volatiles, but they varied greatly in kind and content (Table 1). The volatile compounds found in more than half of the fruits were as follows: α-pinene, limonene, isobutyl acetate, ethyl acetate, isoamyl acetate, 3-methylbutyl butyrate, 3-methyl-1butanol, and 6-methyl-5-hepten-2-one. Half of these are esters, so it can be concluded that esters are commonly found in fruit volatiles. All of the fruits we tested contained terpenoids, except for banana. Esters were the major volatiles in banana, apple, and watermelon, while terpenoids dominated the orange volatiles, which also contained very small amounts of aromatics, such as 1-methyl-4-(1-methylethenyl)-benzene. Persimmon contained a high relative concentration of 3-methyl-4-oxo-pentanoic acid, about 49.36%. In contrast, esters and nitrogen compounds were predominant in pears, which also contained a small amount of hydrocarbons, such as nonane and 1-nonene.

**Figure 3.**
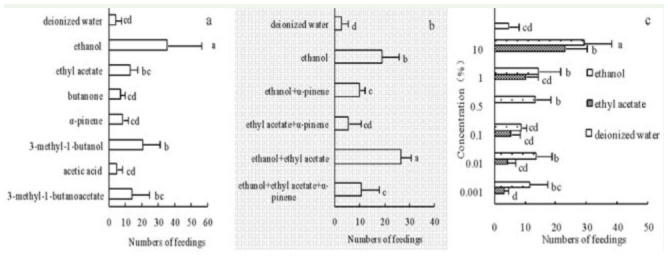
The feeding responses of *Kallima inachus* to different volatiles. A: single compounds at concentrations of 5%. B: compound mixtures at a total concentration of 5%. C: different doses of ethanol and ethyl acetate (mean ± SD). Different letters indicate significant differences with the LSD test (*p* < 0.05). High quality figures are available online.

### Behavioral responses to different volatiles

Single-compound bioassays of seven chemicals at 5% concentration were performed. The number of adult butterflies attracted to these compounds was in the following order, from most attractive to least: ethanol > 3-methyl-1butanol > isoamyl acetate > ethyl acetate > αpinene > butanone > acetic acid > deionized water (Figure 3 a). All seven compounds were attractive to *K. inachus*; however, ethanol was significantly more attractive than the other compounds or deionized water (*p* < 0.05). 3-methyl-1-butanol was also significantly more attractive than deionized water (*p* < 0.05). Although the feedings of isoamyl acetate and ethyl acetate were greater than deionized water, there were no differences (*p* > 0.05). Butanone, α-pinene, and acetic acid were slightly more attractive than deionized water, but there were no significant differences between them (*p* > 0.05).

The synergistic effects of different compound mixtures were also tested. Adults were attracted to these mixtures in the order from most to least, as follows: ethanol + ethyl acetate > ethanol > ethanol + ethyl acetate + α-pinene > ethanol + α-pinene > ethyl acetate + α-pinene > deionized water (Figure 3b). Ethanol + ethyl acetate was the most effective of all mixtures. Overall, the mixtures containing ethanol were significantly more attractive than deionized water (*p* < 0.05), while ethyl acetate + αpinene, the only combination without ethanol, showed a slightly greater lure than deionized water, but showed no differences (*p* > 0.05). The presence of α-pinene reduced the number of feedings.

The effective doses of ethanol and of ethyl acetate were evaluated. The number of responses was significantly positively correlated with concentration for both compounds (r = 0.974, *p* < 0.01; r = 0.976, *p* < 0.01; respectively). For ethyl acetate, only the 10% dose showed significantly higher attractiveness than deionized water (*p* < 0.05), but there were no differences between 0.01%, 0.001% ethyl acetate, and deionized water (*p* > 0.05). Ethanol was more attractive than deionized water regardless of its concentration. Overall, 10% ethanol was the most effective lure, and concentrations of 10%, 1%, 0.5%, and 0.01% were significantly more attractive than deionized water (*p* < 0.05), but there were no differences between concentrations of 0.1%, 0.001%, and deionized water (*p* > 0.05). In addition, ethanol was more attractive than ethyl acetate at the same concentration (Figure 3c).

**Figure 4.**
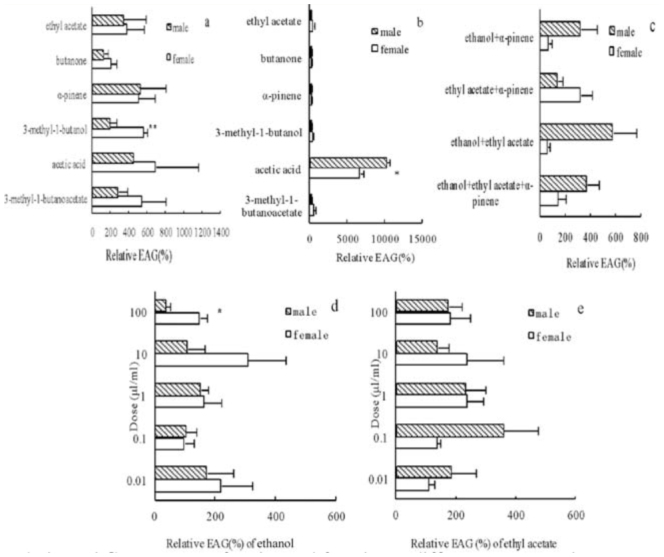
The relative electroantennogram responses of males and females to different compounds, a: reponses to compunds at the dose of 5 µl/mL b: responses to compounds at the dose of 50 µl/mL c: responses to mixtures at the dose of 50 µl/mL d: responses to different doses of ethanol. e: responses to different doses of ethyl acetate (mean ± SE). Significant differences by the paired t-test are indicated by * (*p* < 0.05) and ** (*p* < 0.01). High quality figures are available online.

### EAG recording

Females responded significantly more than males to 3-methyl-1-butanol at 5 µl/mL (*p* < 0.01); however, there were no differences in response between genders in ethyl acetate, butanone, α-pinene, acetic acid, or isoamyl acetate at this concentration (*p* > 0.05) (Figure 4a). At the dose of 50 µl/mL, acetic acid evoked the highest response, and the response value of males was significantly higher than females (*p* < 0.05), but there was no difference in gender-response to the other five compounds at this concentration (*p* > 0.05) (Figure 4b).

The within-sex responses to the six different chemicals were not significantly different at the 5 µl/mL dose (Figure 4a) or at the 50 µl/mL dose (Figure 4b) (*p* > 0.05), indicating that the perception of different compoundswas varied, but the differences were not significant. Ethanol, which was used as a standard, elicited the lowest response in both sexes. EAG responses of both sexes to six chemicals at the dose of 50 µl/mL were weaker than at 5 µl/mL, except for female and male responses to acetic acid, male responses to butanone, and female responses to ethyl acetate, which were somewhat greater than at 5 µl/mL.

There was no difference between the sexes in response to each chemical mixture at the dose of 50 µl/mL (*p* > 0.05) (Figure 4c). For females, the response to ethyl acetate + α-pinene was significantly higher than the responses the other three mixtures (*p* < 0.05). There was no difference in the EAG response of males to the different mixtures (*p* > 0.05).

In the tested concentrations, there were no differences between genders in EAG responses to different doses of ethanol (Figure 4d) and ethyl acetate (Figure 4e) (*p* > 0.05), except for 100 µl/mL ethanol (*p* < 0.05). Different doses of ethanol and ethyl acetate also did not elicit significantly different responses within the sexes (*p* > 0.05) (Figure5d, e, respectively). The values of EAG responses were not correlated to the doses of chemicals (ethanol: females r = -0.194, *p* > 0.05, males r = -0.852, *p* > 0.05; ethyl acetate: females r = 0.071, *p* > 0.05, males r = -0.119, *p* > 0.05).

## Discussion

Alcohols, ketones, and esters were found in all six fruit volatiles, either as the majority components or at lower levels. Esters were the main volatiles in most of the fruits tested. Ethanol and acetic acid, both of which are characteristic volatile products of fermentation (Ômura et al. 2000; Ômura and Honda 2003, 2009), were not found in our samples.

This result may be because the fruit was not thoroughly fermented. Ripe fruits are high in aromatic volatiles, rather than producing decay odor (Molleman et al. 2005). The six kinds of fruit were fermented under the same conditions, so the variations in volatile composition were most likely due to differences in the fruits themselves and to different microbial species associated with the juice extracts (Wood 1961; Ômura et al. 2000).

Although there was no ethanol or acetic acid in the fruit volatiles, the fruits emitted a variety of different types of chemicals and were strongly attractive to the butterfly, indicating that *K. inachus* uses a variety of food chemical signals.

In behavioral tests, alcohols were the most attractive, followed by esters; α-pinene, butanone, and acetic acid were less attractive. Although acetic acid is one of the characteristic volatiles widely found in fermented food, it was not strongly attractive to *K. inachus* at the dose of 5% in the behavioral tests, suggesting acetic acid by itself is not very attractive. In contrast, alcohol is very attractive to *K. inachus*, as 0.01% ethanol was enough to be perceived, and the attraction increased with increasing concentrations. Previous studies (Ômura et al. 2000; Ômura and Honda 2009) have shown that fermentation products such as ethanol and acetic acid elicit intermediate to high proboscis extension reflex responses, but not feeding responses, in *N. xanthomelas, Kaniska canace*, and *V. indica* by synergistically stimulating butterfly feeding behavior when combined with sugars at low concentrations (Ômura and Honda 2003). In our experiment, even though sugar was not used, ethanol and 3-methyl-1-butanol still elicited strong continuous feeding responses in *K. inachus*. In the tropical butterfly *B. anynana*, only sugars and alcohols such as ethanol, butanol, and propanol acted as feeding stimuli and as long range signals to guide the adults to food; various other compounds (e.g. amino acids, acetic acid, vitamins, lipids, salts, and yeast) did not elicit any probing or feeding responses (Dierks and Fischer 2008). Our results showed that alcohols are not only the priority foraging signal, but also elicit feeding responses. Acetic acid showed almost no attractiveness, and esters had a certain lure.

In behavioral tests, ethanol was the most attractive volatile, while acetic acid was the least attractive. In contrast, the perception of antennae to acetic acid in EAG tests was the strongest, while ethanol was the weakest, regardless of whether the test dose was 5 or 50 µl/mL. This result showed acetic acid induces the strongest perception by antenna, but *K. inachus* showed the strongest preference for ethanol. Thus, the compound that most strongly stimulated feeding behavior did not induce high EAG responses, and EAG responses did not totally reflect the food choices of adults foraging in the field (Honda et al. 1998; Ômura and Honda 2009). This interesting result suggests that antennal perception of these olfactory cues was caused by a specific sensory system that selectively elicited feeding behavior (Ômura and Honda 2009). Antennal perception is not only related to foraging behavior, but also involves a large number of other behavioral functions. Therefore, the antennal perception of volatiles is not necessarily interpreted as a food signal; some signals may relate to host recognition, to the identification of conspecific individuals, or to defense (Kreher et al 2008).

Another striking result of the EAG experiments was the differences found between genders for a few chemicals, such as 5 µl/mL butanone, 50 µl/mL acetic acid, and 100 µl/mL ethanol. These differences may be due to males and females differing in their dietary needs because of different reproductive strategies (Dierks and Fischer 2008). However, these differences were not consistent, as they changed at different volatile concentrations. Different concentrations of the same compounds may reflect the distance between adult butterflies and food, but the relative EAG responses of males and females to different concentrations of ethanol and ethyl acetate showed no significant correlation. The phenomenon may be caused by the tested concentration, which could have been higher than the perception threshold of the antenna of *K. inachus*.

In nature, *K. inachus* live mainly in tropical and subtropical areas and are active in broad-leaved forests and forest edges at altitudes of 500–1200 m.a.s.l., where they feed primarily on exuded tree sap and rotting fruits. Although Igarashi and Fukuda (2000) reported that adult *K. inachus* occasionally visit flowers, we have not seen this phenomenon in many years of fieldwork. *K. inachus* are always found in shadowy, hidden habitats, and their larval host plants grow mainly in humid environments. Because of these ecological and biological characteristics, *K. inachus* might prefer food resources within the forest rather than at the forest edge. Before humans significantly impacted the forests, butterfly habitat may not have been as scattered and fragmented as it is today, and large areas of continuous forest habitat may have lacked substantial floral resources. Thus, preferences for sap and rotting fruits as food may have been an important adaptation to a habitat with fewer flowers. Similar phenomena were also found in *Kaniska canace* and *V. indica* (Ômura et al. 2000).

The visual information about food sources available to adults is limited by the weak light inside the forest and by the lack of color contrast between food and its surrounding environment. Leaves, weeds, and other debris could cover food, and the color and shape of rotting food can change dramatically in the decay process. Thus, visual cues may not provide reliable information for foraging adults. In our study, color was not a foraging cue in *K. inachus* and did not stimulate feeding behavior, suggesting there were no innate color preferences in *K. inachus*. However, fermented fruit juices stimulated a strong feeding behavior, supporting the view of Ômura et al. (2000) that odors serve as the pivotal cues in the foraging of fruit-feeding butterflies. We conclude that *K. inachus* give much greater weight to olfactory cues than to visual ones.

*K. inachus* preferred white flowers to the other three colors when fermented odors were present, but their responses to red, yellow, and purple flowers were not significantly different (*p* > 0.05). Because adult *K. inachus* habitually perch on white objects in net houses, their preference for white flowers in our experiment may be due to the background color. White is strongly reflective and may be more beneficial for regulating body temperature. Nocturnal moths, which use olfaction rather than vision to find and recognize nectar resources (Balkenius et al. 2006), have similar characteristics, mainly visiting white, creamcolored, or bright yellow flowers (Wyatt 1983) that offer a strong intensity contrast to the green vegetation or the dark night sky (Kelber et al. 2003). There is considerable evidence that moths are ancestral to butterflies, and the eyes of butterflies evolved from the eyes of moths (Stavenga and Arikawa 2006). *K. inachus* is a highly derived species within the butterfly clade (Wahlberg et al. 2005), so why does *K. inachus* exhibit the ancestral color preferences of moths in its foraging? This question needs further study.

According to the results of our study, we conclude the following: 1) the foraging, adult *K. inachus* has no sensitivity to food color, but innately uses olfaction to detect and locate food; 2) the adults feed on a wide range of fruits, so they also use a wide range of volatiles such as alcohols, esters, ketones, and terpenoids, and the coexistence of multiple chemical signals is most attractive; 3) antennal perception is not only related to foraging behavior, but also involves a large number of other behavioral functions, such as hostrecognition and defense behavior, so feeding preference is not necessary correlated with EAG responses.

**Table 1.**
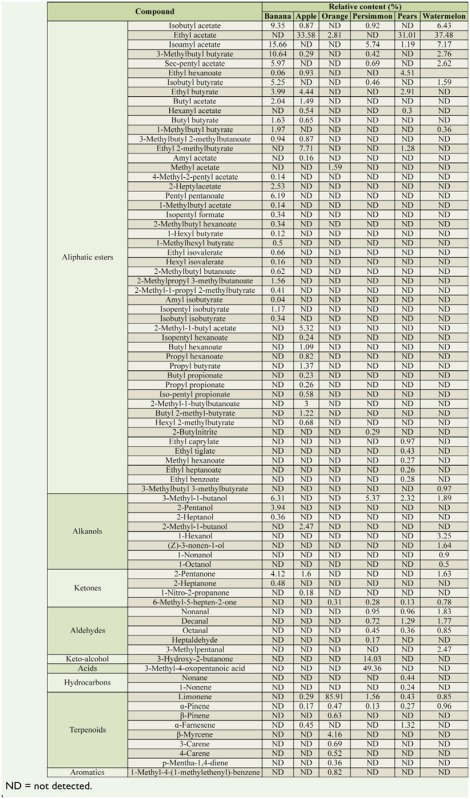
Chemical components of six kinds of fermented fruits and their relative contents (%).
